# Design of Ultra-Thin PEO/PDMAEMA Polymer Coatings for Tunable Protein Adsorption

**DOI:** 10.3390/polym12030660

**Published:** 2020-03-15

**Authors:** Anna Bratek-Skicki

**Affiliations:** 1 Institute of Condensed Matter and Nanosciences, Université catholique de Louvain, Place Louis Pasteur 1, bte L4.01.10, B-1348 Louvain-la-Neuve, Belgium; ncbratek@cyf-kr.edu.pl; 2 Jerzy Haber Institute of Catalysis and Surface Chemistry, Polish Academy of Sciences, Niezapominajek 8, PL30239 Krakow, Poland

**Keywords:** stimuli-responsive coatings, protein adsorption, smart coatings

## Abstract

Protein adsorption on solid surfaces provides either beneficial or adverse outcomes, depending on the application. Therefore, the desire to predict, control, and regulate protein adsorption on different surfaces is a major concern in the field of biomaterials. The most widely used surface modification approach to prevent or limit protein adsorption is based on the use of poly (ethylene oxide) (PEO). On the other hand, the amount of protein adsorbed on poly(2-(dimethylamine)ethyl methacrylate) (PDMAEMA) coatings can be regulated by the pH and ionic strength of the medium. In this work, ultra-thin PEO/PDMAEMA coatings were designed from solutions with different ratios of PEO to PDMAEMA, and different molar masses of PEO, to reversibly adsorb and desorb human serum albumin (HSA), human fibrinogen (Fb), lysozyme (Lys), and avidine (Av), four very different proteins in terms of size, shape, and isoelectric points. X-ray photoelectron spectroscopy (XPS), quartz crystal microbalance (QCM), and atomic force microscopy (AFM) were used to characterize the mixed polymer coatings, revealing the presence of both polymers in the layers, in variable proportions according to the chosen parameters. Protein adsorption at pH 7.4 and salt concentrations of 10^−3^ M was monitored by QCM. Lys and Av did not adsorb on the homo-coatings and the mixed coatings. The amount of HSA and Fb adsorbed decreased with increasing the PEO ratio or its molar mass in a grafting solution. It was demonstrated that HSA and Fb, which were adsorbed at pH 7.4 and at an ionic strength of 10^−3^ M, can be fully desorbed by rinsing with a sodium chloride solution at pH 9.0 and ionic strength 0.15 M from the mixed PEO5/PDMAEMA coatings with PEO/PDMAEMA mass ratios of 70/30, and 50/50, respectively. The results demonstrate that mixed PEO/PDMAEMA coatings allow protein adsorption to be finely tuned on solid surfaces.

## 1. Introduction

Adsorption of proteins at solid surfaces and their interactions are major concerns in many fields such as medicine, biology, biomaterials, biotechnology and plays an important role in a system’s performance [1]. For example, blood contact with a biomaterial initiates rapid adsorption of plasma proteins, which often elicits the foreign body reaction heralded by a massive inflammatory response [2]. On the other hand, an adsorbed protein layer on biomaterials regulates a variety of cell behaviors such as attachment, spreading, proliferation, migration, and differentiation [3]. From a chemical point of view, proteins are the most structurally complex and functionally sophisticated molecules known [4]. Due to their amphiphilic properties, they are intrinsically surface-active molecules; thusly, the problem is not how to adsorb them to interfaces, but how to control their interfacial adsorption. The adsorbing molecules are large, and, thus, the surface-protein interactions are usually long range and include Coulombic forces, van der Waals forces, Lewis acid-base forces, and more entropically based effects such as hydrophobic interactions, conformational entropy and restricted mobilities. Furthermore, due to the large size and the shape of the molecules, the interactions between them on the surface are nontrivial and can be strongly influenced by the fact that the particles may undergo conformational changes upon adsorption [5,6].

Many different strategies have recently been developed to partially or temporally control protein adsorption, mostly based on polymer modified surfaces [7,8]. Polymer layers made from responsive coatings are especially relevant for biological and biosensing applications [9] because external stimuli such as pH, ionic strength or temperature can switch the coatings between, at least, two states tuning their surface properties [10,11,12,13,14,15,16,17]. Coatings composed of weak polyelectrolytes are especially interesting due to their sensitivity to pH because the charge of the chains depends on the protonation/deprotonation of their ionic groups [13]. The interactions of proteins with charged surfaces and their adsorption are mainly regulated by electrostatic interactions but they can nonspecifically adsorb to charged surfaces, regardless of the protein’s net charge [18].

Polycationic coatings consisting of poly (2-dimethylamino ethyl methacrylate) (PDMAEMA) are widely studied in the literature. PDMAEMA is a weak cationic polyelectrolyte in aqueous solutions. It has been found that electrostatic repulsions between the protonated tertiary amine groups lead to PDMAEMA swelling at low pH values. In contrast, at high pH values, most of the amine groups are deprotonated and neutral, which leads to a more compact PDMAEMA conformation. The increase of ionic strength also leads to a collapsed conformation of PDMAEMA as a result of charge screening in the protonated polymer [19]. The formation of polymer layers composed of two different polymers leads to the formation of mixed polymer coatings that might switch individually to external stimulus, and, thusly, a smart surface with a response(s) to the environment can be achieved [20,21,22,23].

Kusumo et al. [24] monitored the binding of bovine serum albumin (BSA) and lysozyme (Lys) to cationic poly(2-(dimethylamino)ethyl methacrylate) (PDMAEMA) coatings grafted onto gold surfaces as a function of the chain length and grafting density. The expected adsorption was observed for negatively charged BSA with a very high binding capacity. However, due to electrostatic repulsion, positively charged Lys was rejected by PDMAEMA coatings. Complete repulsion of proteins by surfaces having the same charge was not always observed. The authors reported a complete lack of BSA desorption from the PDMAEMA coatings when rinsing with diluted NaCl solutions.

Lei et al. [25] studied lysozyme adsorption on silicon surfaces modified by a poly(2-(dimethylamino) ethyl methacrylate)-block-poly (methacrylic acid) (PDMAEMA-b-PMMA) diblock copolymer and both polyelectrolytes separately in a pH range of 4-10. The authors demonstrated that Lys adsorption was low on PDMAEMA coatings and high on PMMA coatings over the studied pH range. Adsorption on PDMAEMA-b-PMMA diblock copolymer coatings showed the influence of both polyelectrolytes. At low pH, the adsorption was low, while at high pH, the adsorption increased with increasing pH and increasing thickness of the PMMA block. When the thickness was greater than 10 nm, the Lys-resistance properties of PDMAEMA were screened, and the diblock copolymer exhibited adsorption similar to the PMMA homopolymer coating. However, Lys desorption from the homo and diblock was not studied.

Wang et al. [26] studied fluorescently labelled BSA adsorption/desorption on PDMAEMA coatings with sub-100 nm features over large areas. The patterned polymer coatings were formed by a combination of block copolymer micelle lithography and surface-initiated atom transfer radical polymerization. The authors confirmed significant adsorption of BSA at pH 5.8. After desorption performed at pH 4, pH 9 and 1 M NaCl only 53 % of proteins were released from the surface.

As it was recently shown, mixed polymer coatings composed of protein-repellent and protein-adsorbing polymers are very promising due to their ability to control protein adsorption/desorption processes [27,28,29,30,31]. For example, Välimäki et al. [31] used poly (ethylene glycol) PEO-PDMAEMA block copolymers for efficient heparin binding. The authors reported two optimized polymers (PEO_114_PDMAEMA_52_ and PEO_114_PDMAEMA_100_) that can neutralize heparin in a dose-dependent manner. The purpose of using PEO was to regulate the amount of heparin. These complexes, due to applying non-toxic PEO, had only a limited effect on cell viability.

This work will bring more insight into the rational design of such smart coatings by introducing, except for PEO, a cationic polymer, poly (2-(dimethylamine) ethyl methacrylate) (PDMAEMA) to adsorb and desorb proteins in a reversible and repetitive way. The effectiveness of protein adsorption/desorption was investigated in terms of polymer coating composition, pH and ionic strength.

Mixed PEO/PDMAEMA polymer coatings were formed on a gold substrate according to the “grafting to” method [32]. The polymer layer was formed by simultaneously grafting PEO and PDMAEMA to the gold substrate. The properties of the mixed polymer coatings were adjusted by the ratio of PEO/PDMAEMA and the PEO chain length. Four proteins, human serum albumin (HSA), human fibrinogen (Fb), lysozyme (Lys), and avidine (Av), all very different from each other, were chosen to study their adsorption on PEO/PDMAEMA coatings.

HSA is the most abundant protein in human plasma and plays a very important role in many processes such as osmotic pressure regulation, the transport of fatty acids, drugs, metals, etc. The protein is globular with a molecular mass of 66 kDa and an iep close to 5.0 [33,34]. Fibrinogen is also a very important protein that is responsible for the regulation of thrombosis, and hemeostasis. It is a linear molecule with a molecular mass of 340 kDa and an iep at pH 5.8. This protein was chosen due to its ability to adsorb below and above its iep, as reported in the literature [30,35,36,37,38]. The third protein chosen was Lys, a small protein (14.3 kDa), with its iep at 11 [25,39,40]. The last protein was Av, a fascinating protein because of its high binding affinity for the vitamin biotin. The molecule is a tetramer composed of four glycosylated subunits having a molecular mass of about 68 kDa. It is a small molecule (4 nm) with iep at pH 10.5 [41,42]. The adsorption of the proteins was performed at pH 7.4 and *I* = 10^−3^ M.

## 2. Experimental Section

### 2.1. Materials 

Gold substrates for X-ray photoelecton spectroscopy (XPS) were prepared by the thermal evaporation of gold (thickness of 100 nm) onto silicon wafers with a titanium interlayer. Next, they were cut into pieces approximately 1 cm^2^ in size. Before every measurement, the gold substrates, and QCM quartz sensors coated with a 100 nm Au layer, were cleaned in a mixture of 95% sulfuric acid (H_2_SO_4_, VWR BDH Prolabo, Leuven, Belgium) and hydrogen peroxide (30%, VWR BDH Prolabo) in a volume ratio of 2:1 for 2 min. In the next step, the gold substrate was rinsed 10 times with deionized water, once with absolute ethanol, and dried out in a stream of nitrogen gas. Afterwards, the gold was exposed in a UV/ozone environment (Jelight Inc., Irvine, CA, USA) for 15 min. In the end, the substrate was rinsed again with absolute ethanol and dried using a nitrogen flow.

**Figure 1:**
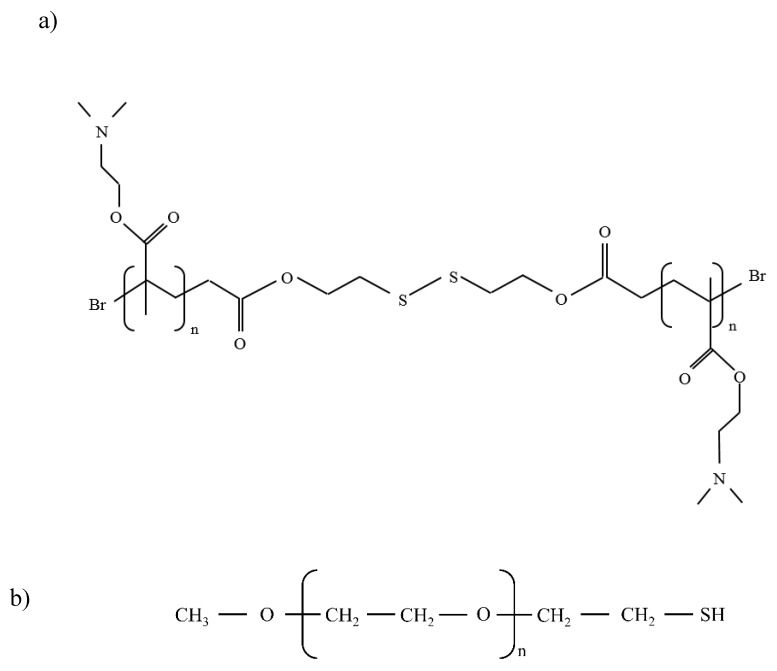
Chemical structure of PDMAEMA (**a**) and PEO (PEO1, n = 23 units, PEO2, 43 units, PEO5, n = 112 units) (**b**).

The polymers containing the thiol groups were purchased from Polymer Source Inc. (Dorval, QC, Canada). Poly (2-(dimethylamine) ethyl methacrylate) (PDMAEMA) with a disulfide bond had a molar mass of *M_n_* = 8500 g/mol, 2 × 28 repeating units, and a polydispersity index of 1.30 (see Figure 1a). Thiolated poly (ethylene glycol) methyl ether was used with three molar masses: *M_n_* = 1100 g/mol (~23 units-PEO1), *M_n_* = 2000 g/mol (~43 units-PEO2), *M_n_* = 5000 g/mol (~112 units-PEO5) (see Figure 1b), and polydispersity indices of 1.08, 1.09, and 1.08, respectively. Stock solutions of PDMAEMA and PEO (PEO1, PEO2, PEO 5) were prepared in ultrapure water at a concentration of 3 and 5 g/L, respectively. Before each experiment, they were diluted in water to the desired concentration of 1g/L. Formation of polymer coatings was conducted by the immersion of cleaned gold substrates in mixtures of PEO and PDMAEMA solutions, with PEO/PDMAEMA mass ratios of 100/0 (PEO), 50/50 (PEO/PDAEMA 50/50), 60/40 (PEO/PDMAEMA 60/40), 70/30 (PEO/PDMAEMA 70/30) and 0/100 (PDMAEMA).

### 2.2. Methods

#### 2.2.1. X-Ray Photoelectron Spectroscopy (XPS)

Gold substrates with anchored polymers were analyzed using a Kratos Axis Ultra spectrometer (Kratos, Analytical, Manchaster, UK) with an Al X-ray source. The samples were placed on an insulating ceramic holder and placed inside a chamber having a pressure of 10^−6^ Pa. The angle between the sample and the analyzer was 55°. Charge stabilization was achieved by applying a flood gun at 8 eV and a Ni grid was placed 3 mm above the analyzed sample. The applied pass energy was 150 eV and the following spectra was collected: survey spectrum, C 1s, O 1s, N 1s, S 2p, Au 4f and C 1s. After this step the stability of the charged compensation and degradation of the samples were checked. The characteristic peak corresponding to the binding energy of C–(C, H) was fixed at 284.8 eV. The obtained data was analyzed using the CasaXPS Program (Casa Software Ltd., Teignmouth, UK).

#### 2.2.2. Quartz Crystal Microbalance with Dissipation Monitoring (QCM-D)

**Figure 2:**
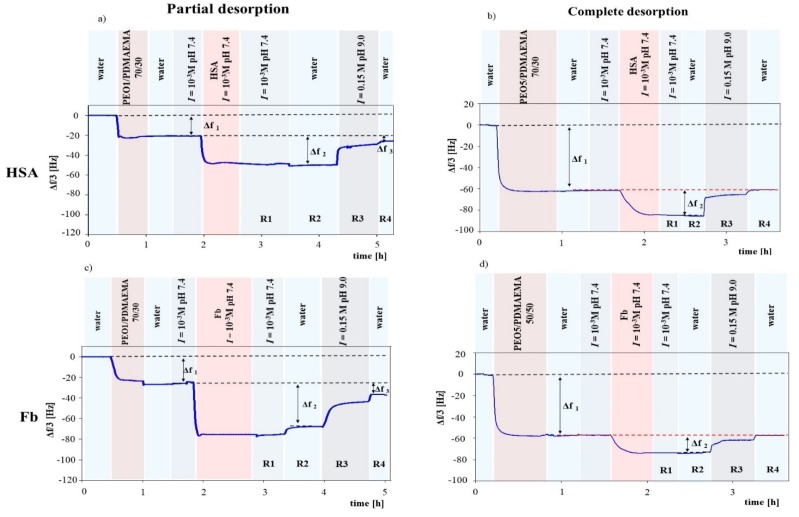
QCM graph of PEO/PDMAEMA mixed coatings formed on a gold surface followed by steps of adsorption/desorption of proteins: (**a**) HSA adsorption on PEO1/PDMAEMA 70/30, (**b**) HSA adsorption on PEO5/PDMAEMA 70/30 coatings, (**c**) Fb adsorption on PEO1/PDMAEMA 70/30, (**d**) Fb adsorption on PEO5/PDMAEMA 50/50 coatings (adsorption at pH 7.4 and *I* = 10^−3^ M, desorption at *I* = 0.15 M, pH 9.0), R1- rinsing with the saline solution of the same pH and ionic strength as used for protein adsorption, R2 - rinsing with ultrapure water, R3 - introduction of a saline solution of 0.15 M and pH 9.0, R4 - rinsing with ultrapure water.

Formation of polymeric layers was monitored by Quartz Crystal Microbalance (Q-Sense E4 System, Q-Sense, Gothenborg, Sweden) at 298 K using gold quartz sensors. At the beginning of each measurement ultrapure water was flowed into a cell until a stable baseline was obtained. Next, the polymer solution (PEO, PDMAEMA, PEO/PDMAEMA 50/50, PEO/PAA 60/40 or PEO/PAA 70/30) was introduced into the cell with a flow rate of 20 μL/min. When stable signals (frequency and dissipation) were obtained, the cell was rinsed with ultrapure water in order to remove unbound polymer molecules with a flow rate of 50 μL/min. Afterwards, a solution of *I* = 10^−3^ M and pH = 7.4 (flow rate = 50 μL/min) was flowed into the cell to test the stimuli-responsive behavior. In the next step, the protein solution, having a concentration of 0.2 mg/mL, was introduced to the system with a flow rate of 20 μL/min. After obtaining a stable signal, the flow with the protein solution was stopped and rinsed with the same saline solution (*I* = 10^−3^ M and pH = 7.4, see Figure 2, R1) and ultrapure water (R2). The final desorption step consisted of the following steps: introduction of 0.15 M NaCl, pH 9.0 (R3), and rinsing with ultrapure water (R4) with a flow rate of 50 μL/min.

#### 2.2.3. Atomic Force Microscopy (AFM)

AFM images were acquired with a Nanoscope III instrument (Digital Instruments, Santa Barbara, CA, USA) operated in tapping mode. Non-conductive silicon tips, having a spring constant between 10 and 130 N/m and resonance frequency 204–497 kHz (Nanosensors, Neuchâtel, Switzerland) were used. The scan rate was 1 Hz, and the image size was 2 × 1 µm^2^. Next, the images were plane-fitted and flattened using the Gwyddion software (an open source software), http://gwyddion.net/.

## 3. Results and Discussion

### 3.1. PEO and PDMAEMA Homo-Coating Characterization

**Figure 3:**
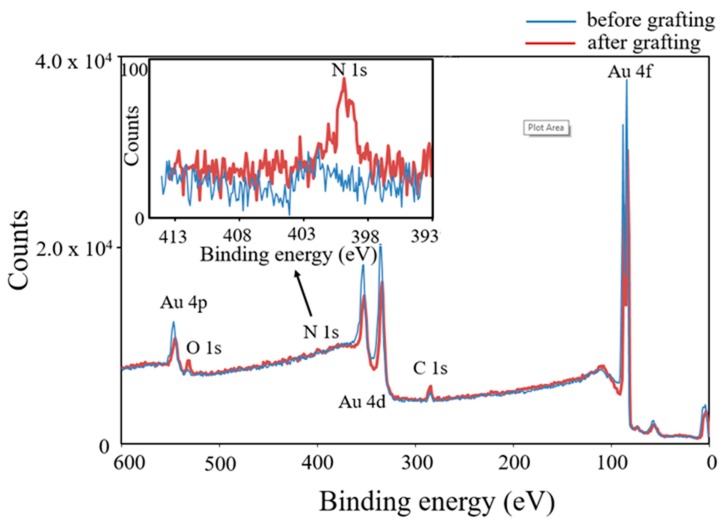
XPS spectra of gold surface before and after grafting with poly (2-(dimethylamino) ethyl methacrylate) (PDMAEMA). Inset: enlarged XPS spectra of N 1s region. N 1s signal appears after PDMAEMA grafting.

**Figure 4:**
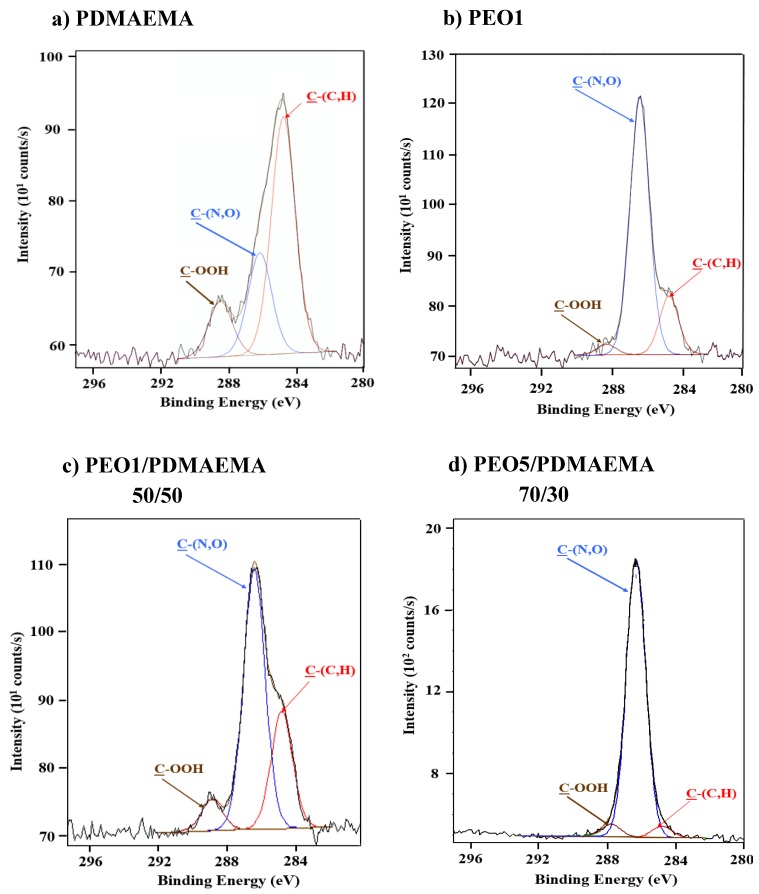
C 1s peaks recorded by XPS on gold surfaces modified with thiol-functionalized polymers: (**a**) PDMAEMA, (**b**) PEO 1, (**c**) PEO1/PDMAEMA 50/50, (**d**) PEO1/PDMAEMA 70/30. Peak decomposition was performed according to the protocol described in the Section S1.

The surface composition of the gold substrate before and after grafting with PEO and PDMAEMA was studied by X-ray photoelectron spectroscopy. After grafting PDMAEMA to the gold substrate, the N 1s signal ~399 eV (see Figure 3) was recorded which corresponds to the dimethylamino group. The intensity of Au 4f signals at ~84.5 eV and ~88.5 eV decreased. This measurement confirmed the presence of PDMAEMA on the gold surface. Figure 4a,b illustrates the C 1s peak recorded by XPS on a gold surface modified with PDMAEMA and PEO1, respectively.

The method of the C 1s peak decomposition was described in Section S1 (supplementary material). It can be noticed that in Figure 4a the **C**–(C, H) component at 284.8 eV attributed to the –CH_3,_ –CH_2_–, groups of PDMAEMA is dominating the spectrum. The respective presence of components at 286.3 eV attributed to **C**–(N, O) and at 288.8 eV corresponding to **C**=O also confirms the presence of PDMAEMA. In Figure 4b, it can be observed that the **C**–O–**C** component attributed to PEO is much larger in comparison to Figure 4a. However, there is also a small **C**=O component attributed to contaminants. Surface atomic fraction (%) of component at 286.3 eV attributed to **C**–O–**C** after grafting PEO1, PEO2 and PEO5 to the gold substrate is presented in Table S1. The **C**–O–**C ** fraction attributed to PEO increased with increasing PEO molar mass from 25.5 to 46 for PEO1 and PEO5, respectively.

The polymer coating formation was also monitored in situ using quartz crystal microbalance with dissipation monitoring. A typical QCM polymer coating formation experiment is presented in Figure 2. After stabilizing the frequency signal in water, the polymer solution (PDMAEMA, PEO, mixture of PEO/PDMAEMA) was flowed into the cell, allowing a frequency shift to be recorded. The frequency shift *Δf_1_* stabilized during the rinsing with water. The corresponding wet mass of polymer (Δ*m*) was calculated using the Sauerbrey relation and is presented in Table 1. The wet mass of PDMAEMA was 505 ng/cm^2^, while for pure PEO coatings it increased with increasing their molar mass from 547 to 1195 ng/cm^2^, respectively. Furthermore, taking into account the density of both polymers (PEO: 1.13 g/cm^3^; PDMAEMA:1.32 g/cm^3^) [43], the thickness of the polymer coatings was calculated (Table 1). The estimated thickness of the pure PDMAEMA coating was 3.8 nm, while the thickness of pure PEO coating increased with increasing their molar mass from 4.8 to 10.2 nm for PEO1 and PEO5, respectively.

The significant presence of **C**–(N, O) and **C**=O components proves that PDMAEMA and PEO were successfully grafted to the gold substrate. Moreover, the estimated wet masses of pure PDMAEMA and PEO coatings, as well as their corresponding thicknesses, also confirm successful homopolymer coating formation.

### 3.2. The Mixed PEO/PDMAEMA Coating Characterization: Effect of PEO/PDMAEMA Ratio and PEO Molar Mass

Figure 4c,d presents the C 1s peak recorded by XPS on a gold surface modified by the simultaneous grafting of PEO1/PDMAEMA 50/50 and PEO5/PDMAEMA 70/30, respectively. It can be noticed that in both cases the **C**–(N, O) component at 286.3 eV dominates the spectra. Moreover, the fraction of **C**=O component at 288.8 eV, attributed to PDMAEMA confirms its presence in the mixed coatings. The proportions of both components depend on the PEO/PDMAEMA ratio: a larger **C**–(N, O) peak component and a smaller **C**=O component were recorded when more PEO was introduced in the grafting solution (see Figure 4d vs Figure 4c).

Furthermore, taking into account the surface atomic fractions (%) (see Table S1) characteristic for PEO and PDMAEMA components, volume fractions of PEO and PDMAEMA in the mixed coatings were estimated using a method described in Section S1 (Supplementary Materials). 

In Table 1, it can be observed that for coatings PEO1/PDMAEMA and PEO2/PDMAEMA created in the ratio of 50/50, 60/40, 70/30 the volume fraction of PEO changes between 0.52 to 0.68 while for PDMAEMA, it changes from 0.32 to 0.48. The highest volume fraction of PEO was observed for the mixed PEO5/PDMAEMA coatings and it changed from 0.83 for PEO5/PDMAEMA 50/50 and PEO5/PDMAEMA 60/40 to 0.88 for PEO5/PDMAEMA 70/30. In contrast, the lowest PDMAEMA fraction was found for the same set of coatings and it changed from 0.12 to 0.17 for PEO5/PDMAEMA 70/30 and PEO5/PDMAEMA (60/40 and 50/50), respectively. 

The wet mass of the mixed coatings was also estimated using the Sauerbray relation and is presented in Table 1. The wet mass of polymer coatings increased with increasing the PEO ratio in the grafting solution and PEO molar mass. The lowest values of wet masses were observed for PEO/PDMAEMA 50/50 coatings and it changed from 480 to 910 ng/cm^2^ for PEO1/PDMAEMA and PEO5/PDMAEMA. Next, for the series of PEO/PDMAEMA 60/40 the values varied between 508 to 922 ng/cm^2^ for PEO1/PDMAEMA and PEO5/PDMAEMA. The highest values were observed for the PEO/PDMAEMA 70/30 coatings which were between 511 to 1055 ng/cm^2^ for PEO1/PDMAEMA 50/50 and PEO5/PDMAEMA 70/30, respectively. Furthermore, similarly to homo-coatings, the thickness of the mixed coatings was estimated and presented in Table 1. The thickness increased with increasing the PEO ratio in a grafting solution and PEO molar mass. For PEO/PDMAEMA 50/50, the thickness changed from 3.9 to 7.4 for PEO1/PDMAEMA and PEO5/PDMAEMA. Next, for PEO/PDMAEMA 60/40 it varied from 4.1 to 7.5 nm for PEO1/PDMAEMA and PEO5/PDMAEMA. The highest thickness was observed for the PEO/PDMAEMA 70/30 series with 4.2 nm corresponding to PEO1/PDMAEMA 70/30 and 8.6 nm attributed to PEO5/PDMAEMA 70/30. In order to check the homogeneity of the formed homo- and mixed coatings, atomic force microscopy measurements were performed (Table S2). At the presented images, only gold grains could be observed without any aggregates.

The volume fraction of PEO and PDMAEMA in the mixed coatings was similar for both PEO1/PDMAEMA and PEO2/PDMAEMA coatings. The highest PEO volume fraction was observed for the coatings containing the longest PEO chain (PEO5) and it was above 0.8. The estimated wet masses of mixed PEO/PDMAEMA coatings, as well as their corresponding thicknesses, increased with increasing PEO ratio in grafting solution and PEO molar mass.

### 3.3. Protein Adsorption on the Homo- and Mixed PEO/PDMAEMA Coatings

Protein-repellent properties of PEO coatings were studied using the QCM-D method. As an illustration of the PEO properties, a QCM-D experiment of Fb adsorption on the PEO5 coating was selected and is presented in Figure S1. 

**Figure 5:**
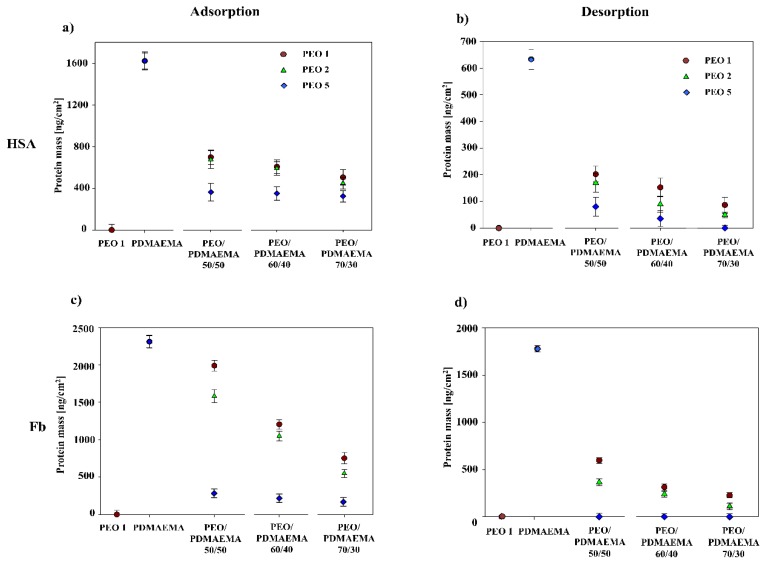
Protein mass obtained from the Sauerbrey modeling of Δ*f* measured by QCM-D after adsorption (left, **a**, **c**) and desorption (right, **b**, **d**) on PEO1, PDMAEMA coatings, and the mixed PEO/PDMAEMA 50/50, PEO/PDMAEMA 60/40, PEO/PDMAEMA 70/30; dark red circles – coatings containing PEO1, green triangles –coatings containing PEO2, blue diamonds- coatings containing PEO5. Top: HSA mass after adsorption at *I* = 10^−3^ M, pH 7.4 and residual mass after desorption (ng/cm^2^) at *I* = 0.15 M, pH 9.0) of HSA, bottom: Fb mass after adsorption at *I* = 10^−3^ M pH 7.4, and residual mass (%) after desorption at *I* = 0.15 M, pH 9.0.

The same experiments were performed with HSA, Lys and Av on coatings formed from PEO1, PEO2, and PEO5. The experimental data showing the repellent properties of PEO1 towards HSA and Fb are presented in Figure 5a,c. Similar results (not shown) were achieved for PEO2 and PEO5 coatings.

Conversely, the pure PDMAEMA coatings adsorbed HSA and Fb (see Figure 5a,c). The average adsorbed mass of HSA at *I* = 10^−3^ M, pH 7.4 was 1622 ng/cm^2^. After desorption, performed at *I* = 0.15 M, pH 9.0, the remaining mass was equal to 633 ng/cm^2^ (see Figure 5b). Regarding Fb adsorption on the pure PDMAEMA coatings at *I* = 10^−3^ M, pH 7.4, the calculated mass of the protein was 2310 ng/cm^2^. Partial desorption of Fb from pure PDMAEMA was also observed and the measured mass of Fb after desorption was 1779 ng/cm^2^.

**Scheme 1:**
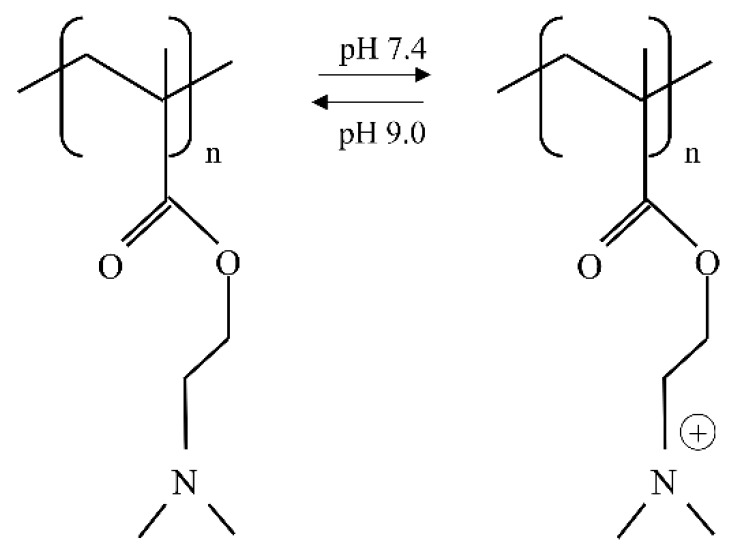
Protonated and deprotonated formulas of PDMAEMA.

At pH 7.4, PDMAEMA is protonated as presented in Scheme 1 and protein adsorption is governed by electrostatic interactions between the positively charged PDMAEMA and negatively charged HSA and Fb. When pH changed from 7.4 to 9.0, deprotonation of dimethyloamino groups takes place and, on the one hand, the decrease of the electrostatic repulsion between PDMAEMA chains causes a collapse of PDMAEMA, and, on the other hand, the decrease in the electrostatic attraction forces between the two proteins and PDMAEMA chains results in partial desorption. Both pH and ionic strength contribute to protein desorption. [22,42] The high ionic strength screens localized electrostatic attractions and provides high concentrations of Cl- as counter ions to exchange the proteins in the coating. The remaining proteins might be trapped in the PDMAEMA chain structures or different forces are still strong enough to keep the proteins on the surface.

It should be noted that Lys and Av adsorption on pure PDMAEMA and the mixed PEO/PDMAEMA coatings was also performed at *I* = 10^−3^ M, pH 7.4. Under these conditions, adsorption of the proteins on the pure and the mixed coatings, due to electrostatic repulsion, was not observed. Two examples of such experiments showing adsorption of Lys and Av on the mixed PEO1/PDMAEMA 70/30 coatings are presented in Figures S2 and S3.

HSA and Fb adsorption experiments were performed on the mixed PEO/PDMAEMA 50/50, PEO/PDMAEMA 60/40 and PDMAEMA 70/30 coatings with PEO having different molar masses (PEO1, PEO2, PEO5). In these experiments *Δf_2_* was also used to estimate protein mass after adsorption and *Δf_3_* was used to calculate the remaining mass after desorption (see Figure 2).

Figure 2a shows a QCM graph of HSA adsorption on the PEO1/PDMAEMA 70/30 coatings. It can be observed that the polymer coating was successfully formed on the gold crystal and the calculated HSA mass after the adsorption step using *Δf_2_* and after rinsing with a 10^−3^ M, pH 7.4 solution (R1) and ultrapure water (R2), was 529 ng/cm^2^. The next two shifts correspond to rinsing with a 0.15 M pH 9.0 saline solution (R3) and ultrapure water (R4). After the desorption step (*Δf_3_*), 87 ng/cm^2^ of HSA remained on the coating. Figure 2b presents HSA adsorption on the PEO5/PDMAEMA 70/30 coating. After the polymer coating formation step (*Δf_1_*), HSA adsorption occurred (*Δf_2_*) with a corresponding mass of 324 ng/cm^2^. In this case, after the desorption step, complete desorption was observed (*Δf_3_ ~* 0). 

Fibrinogen adsorption on PEO1/PDMAEMA 70/30 is presented in Figure 2c. After the adsorption step the calculated mass of Fb was 752 ng/cm^2^, and the remaining protein mass after the adsorption step was 226 ng/cm^2^. In Figure 2d fibrinogen adsorption on PEO5/PDMAEMA 50/50 shows that 264 ng/cm^2^ was deposited on the mixed coating. However, in this case, total desorption of fibrinogen was achieved (*Δf_3_*~0). Adsorption and desorption of HSA and Fb on PEO5/PDMAEMA 70/30 and PEO5/PDMAEMA 50/50 was tested in cycles proving that adsorption and desorption of the proteins can be perform in a reversible and repetitive way (see Figure S4). Total desorption of HSA from PEO5/PDMAEMA 70/30 coatings and Fb from PEO5/PDMAEMA 50/50 coatings proves that PEO plays an important role in the desorption process. Moreover, PEO effectiveness on desorption behavior of both proteins is rather related to the length of the PEO chain than a high content of the PEO having lower masses.

A summary of the results obtained for the adsorption of HSA and Fb on the pure PEO1, PDMAEMA and the mixed PEO/PDMAEMA coatings as a function of PEO ratio in a grafting solution and its molar mass is presented in Figure 5. The highest amount of HSA was observed on the pure PDMAEMA coating (1622 ng/cm^2^) and it decreased while increasing the PEO ratio in a grafting solution or its molar mass (circle PEO1, triangle PEO2, diamond PEO5). For PEO1/PDMAEMA coatings it changed from 698 ng/cm^2^ for PEO1/PDMEMA 50/50 to 529 ng/cm^2^ for PEO1/PDMEMA 70/30. The same tendencies were observed for coatings created from PEO2/PDMAEMA and PEO5/PDMAEMA and the HSA mass changed from 675 ng/cm^2^ for PEO2/PDMAEMA50/50 to 448 ng/cm^2^ for PEO2/PDMAEMA 70/30. 

The lowest HSA mass was observed on PEO5/PDMAEMA coatings and it varied from 364 to 324 ng/cm^2^ for PEO5/PDMAEMA50/50 and PEO5/PDMAEMA 70/30, respectively (Figure 5a). Similarly to HSA, the highest mass of Fb was observed after adsorption on the pure PDMAEMA (2310 ng/cm^2^) and the mass decreased with increasing PEO ratio and its molar mass. For PEO1/PDMAEMA coatings it changed from 1990 ng/cm^2^ for PEO1/PDMAEMA 50/50 to 752 ng/cm^2^ for PEO1/PDMEMA 70/30. Similar changes were also observed for PEO2/PDMAEMA coatings and they varied from 1583 to 550 ng/cm^2^ for PEO2/PDMAEMA50/50 and PEO2/PDMAEMA 70/30, respectively. A more significant decrease in the Fb mass was observed after adsorption on PEO5/PDMAEMA coatings. The Fb mass changed from 264 to 180 ng/cm^2^ for PEO5/PDMAEMA50/50 and PEO5/PDMAEMA70/30 coatings. The results achieved after the desorption experiments of HSA and Fb from the pure PDMAEMA and the mixed PEO/PDMAEMA coatings monitored by the QCM-D are presented in Figure 5 (right panel, b,d).

The lowest HSA mass after desorption was observed for PEO5/PDMAEMA coatings and total desorption was achieved from the PEO5/PDMAEMA 70/30 coating (see Figure 5b). 

A similar dependence was observed for Fb (see Figure 5d). A partial desorption was observed for the pure PDMAEMA and the mixed PEO1/PDMAEMA and PEO2/PDMAEMA coatings. The highest mass of Fb after desorption was measured for the pure PDMAEMA coating (1779 ng/cm^2^) and it decreased while increasing the PEO ratio and its molar mass. For example, for PEO1/PDMAEMA50/50 coatings it was 597 ng/cm^2^ while for PEO1/PDMAEMA70/30 it was 226 ng/cm^2^. Lower Fb mass was observed for PEO2/PDMAEMA coatings and it varied from 364 ng/cm^2^ to 77 ng/cm^2^ for PEO2/PDMAEMA 50/50 and PEO2/PDMAEMA 70/30, respectively. Complete desorption of Fb was achieved from all PEO5/PDMAEMA coatings.

**Figure 6:**
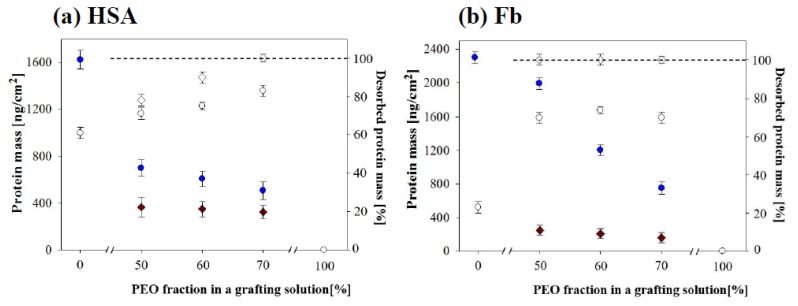
Dependence of protein mass obtained from the Sauerbrey modeling of *Δf* recorded by QCM-D after the adsorption step (left axis, R2, *I* = 10^−3^ M, pH 7.4) and a percentage of remaining protein mass after the desorption step (right axis, R4 *I* = 0.15 M, pH 9.0) as a function of PEO fraction (%) in the grafting solution (PEO1-circles, PEO5-diamonds, closed symbols - protein mass after adsorption, open symbols – protein mass after desorption): (**a**) HSA, (**b**) Fb.

Figure 6 shows the dependence of protein mass (part a for HSA and b for Fb) calculated from the Sauerbrey modeling of *Δf* recorded by QCM-D after the adsorption step (R2, *I* = 10^−3^ M, pH 7.4) and a percentage of desorbed protein mass after the desorption step (R4: *I* = 0.15 M, pH 9.0, water) as a function of PEO fraction (%) (PEO1-circles, PEO5-diamonds, closed symbols-mass after adsorption, open symbols desorption) in the grafting solution. The adsorbed and remaining mass of proteins after desorption decreased with increasing the PEO ratio and its molar mass in the coating. Therefore, it should be noted that protein-repellent PEO plays an important role in both processes. For adsorption, its increasing presence leads to decreased PDMAEMA density in the coating, while exposure of PEO chains upon PDMAEMA shrinking in the desorption process increases protein removal from the coating.

In order to effectively and reversibly adsorb and desorb HSA from the mixed PEO/PDMAEMA coating, the highest molar mass of PEO should be used (PEO5) in the ratio of 70/30 in the grafting solution. For the effective adsorption/desorption of Fb from the mixed PEO/PDMAEMA coatings, the highest PEO molar mass should also be used with the minimum ratio of 50/50. The increasing presence of PEO in the polymer coatings, on the one hand, decreases PDMAEMA density that results in lower amounts of proteins, and on the other hand, its repellent properties followed by shrinking the PDMAEMA chains triggers the desorption processes.

In this section, it was shown that Lys and Av do not adsorb on the pure PDMAEMA and the mixed PEO/PDMAEMA coatings. HSA and Fb, however, adsorbed on the pure PDMAEMA and the mixed PEO/PDMAEMA coatings (50/50, 60/40, 70/30) and the amount of adsorbed proteins decreases with increasing the PEO ratio in the grafting solution or its molar mass. A partial desorption of HSA was observed for the pure PDMAEMA and the mixed PEO1/PDMAEMA, PEO2/PDMAEMA, PEO5/PRDMAEMA 50/50 and PEO5/PDMAEMA 70/30 coatings while its total desorption was achieved from the PEO5/PDMAEMA 70/30 coating. 

Similarly, to HSA, a partial desorption of Fb was observed for the pure PDMAEMA coating and the mixed PEO1/PDMAEMA and PEO2/PDMAEMA coatings while total desorption was observed for all the PEO5/PDMAEMA coatings.

## 4. Conclusions

The presented work shows the effectiveness of the mixed PEO/PDMAEMA coatings to reversibly adsorb and desorb human serum albumin and human fibrinogen. The effect of PEO content in the coating regulated by its ratio in the grafting solution as well as by its molar mass is also discussed. Lack of Lys and Av adsorption on the pure and the mixed coatings is also demonstrated. The amount of HSA and Fb decreases while increasing the PEO content in the grafting solution or its molar mass. Under the presented adsorption and desorption conditions, both processes are mainly governed by the electrostatic interactions between PDMAEMA and the proteins. Desorption is also triggered by the exposure of PEO chains upon PDMAEMA shrinking. An effective adsorption/desorption of HSA can be achieved at the PEO5/PDMAEMA 70/30 coating while the reversible adsorption of Fb can be performed on the PEO5/PDMAEMA 50/50 coating.

## Figures and Tables

**Table 1:** Characterization of Polymer Coatings Formed on Gold Surface.

Sample Name	*V_PEO_*	*V_PDMAEMA_*	*Δm* (ng/cm^2^)	*d* (nm)
Au-PDMAEMA	-	1.0	505 +/− 96	3.8
Au-PEO1	1.0	-	547 +/− 56 *	4.8 *
Au-PEO2	1.0	-	675 +/− 63 *	6.0 *
Au-PEO5	1.0	-	1195 +/− 50 *	10.2 *
Au-PEO1/PDMAEMA 50/50	0.61	0.39	480 +/− 104	3.9
Au-PEO2/PDMAEMA 50/50	0.61	0.39	609 +/− 85	5.0
Au-PEO5/PDMAEMA 50/50	0.83	0.17	910 +/− 94	7.4
Au-PEO1/PDMAEMA 60/40	0.52	0.48	508 +/− 63	4.1
Au-PEO2/PDMAEMA 60/40	0.68	0.32	711 +/− 85	5.8
Au-PEO5/PDMAEMA 60/40	0.83	0.17	922 +/− 63	7.5
Au-PEO1/PDMAEMA 70/30	0.62	0.38	511 +/− 102	4.2
Au-PEO2/PDMAEMA 70/30	0.57	0.43	762 +/− 93	6.2
Au-PEO5/PDMAEMA 70/30	0.88	0.12	1055 +/− 65	8.6

* Ref. [30].

## Supplementary Materials

The following are available online at https://www.mdpi.com/2073-4360/12/3/660/s1.

## References

[1] Nakanishi K., Sakiyama T., Imamura K. (2001). On the adsorption of proteins on solid surfaces, a common but very complicated phenomenon. J. Biosci. Bioeng..

[2] Ombelli M., Costello L., Postle C., Anantharaman V., Meng Q.C., Composto R.J., Eckmann D.M. (2011). Competitive protein adsorption on polysaccharide and hyaluronate modified surfaces. Biofouling.

[3] Webb K., Hlady V., Tresco P.A. (2000). Relationships among cell attachment, spreading, cytoskeletal organization, and migration rate for anchorage-dependent cells on model surfaces. J. Biomed. Mater. Res..

[4] Alberts B., Johnson A., Lewis J., Raff M., Roberts K., Walter P. (2002). Molecular Biology of the Cell.

[5] Hlady V., Buijs J. (1996). Protein adsorption on solid surfaces. Curr. Opin. Biotechnol..

[6] Fang F., Szleifer I. (2001). Kinetics and thermodynamics of protein adsorption: A generalized molecular theoretical approach. Biophys. J..

[7] Wei Q., Becherer T., Angioletti-Uberti S., Dzubiella J., Wischke C., Neffe A.T., Lendlein A., Ballauff M., Haag R. (2014). Protein interactions with polymer coatings and biomaterials. Angew. Chem. Int. Ed..

[8] Wei Q., Haag R. (2015). Universal polymer coatings and their representative biomedical applications. Mater. Horiz..

[9] Kroning A., Furchner A., Aulich D., Bittrich E., Rauch S., Uhlmann P., Eichhorn K.J., Seeber M., Luzinov I., Kilbey S.M. (2015). In Situ Infrared Ellipsometry for Protein Adsorption Studies on Ultrathin Smart Polymer Brushes in Aqueous Environment. ACS Appl. Mater. Interfaces.

[10] Bittrich E., Rodenhausen K.B., Eichhorn K.J., Hofmann T., Schubert M., Stamm M., Uhlmann P. (2010). Protein adsorption on and swelling of polyelectrolyte brushes: A simultaneous ellipsometry-quartz crystal microbalance study. Biointerphases.

[11] Bittrich E., Burkert S., Müller M., Eichhorn K.J., Stamm M., Uhlmann P. (2012). Temperature-sensitive swelling of poly (N-isopropylacrylamide) brushes with low molecular weight and grafting density. Langmuir.

[12] Zhou J., Wang G., Hu J., Lu X., Li J. (2006). Temperature, ionic strength and pH induced electrochemical switching of smart polymer interfaces. Chem. Commun..

[13] Zhang J., Kou R., Liu G. (2017). Effect of salt concentration on the pH responses of strong and weak polyelectrolyte brushes. Langmuir.

[14] Wang W., Li L., Henzler K., Lu Y., Wang J., Han H., Tian Y., Wang Y., Zhou Z., Lotze G. (2017). Protein immobilization onto cationic spherical polyelectrolyte brushes studied by small angle X-ray scattering. Biomacromolecules.

[15] Chen W., Menzel M., Watanabe T., Prucker O., Rühe J., Ober C.K. (2017). Reduced lateral confinement and its effect on stability in patterned strong polyelectrolyte brushes. Langmuir.

[16] Mu B., Liu P., Li X., Du P., Dong Y., Wang Y. (2012). Fabrication of flocculation-resistant pH/ionic strength/temperature multiresponsive hollow microspheres and their controlled release. Mol Pharm.

[17] Moya S., Azzaroni O., Farhan T., Osborne V.L., Huck W.T. (2005). Locking and unlocking of polyelectrolyte brushes: Toward the fabrication of chemically controlled nanoactuators. Angew. Chem. Int. Ed..

[18] Schlick T. (2012). Innovation in Biomolecular Modelling and Simulations.

[19] Su Y., Sun M., Wang L., Jiang Z. (2009). Ion-pair formation and ion-specific flux of a weak polyelectrolyte membrane. J. Phys. Chem. B.

[20] Li L., Nakaji-Hirabayashi T., Kitano H., Ohno K., Saruwatari Y., Matsuoka K. (2017). A novel approach for UV-patterning with binary polymer brushes. Colloids Surf. B Biointerfaces.

[21] Psarra E., König U., Ueda Y., Bellmann C., Janke A., Bittrich E., Eichhorn K.J., Uhlmann P. (2015). Nanostructured biointerfaces: Nanoarchitectonics of thermoresponsive polymer brushes impact protein adsorption and cell adhesion. ACS Appl. Mater. Interfaces.

[22] Xu F., Li H., Li J., Teo Y., Zhu C., Kang E., Neoh K.G. (2008). Spatially well-defined binary brushes of poly (ethylene glycol)s for micropatterning of active proteins on anti-fouling surfaces. Biosens. Bioelectron.

[23] Uhlmann P., Houbenov N., Brenner N., Grundke K., Burkert S., Stamm M. (2007). In-situ investigation of the adsorption of globular model proteins on stimuli-responsive binary polyelectrolyte brushes. Langmuir.

[24] Kusumo A., Bombalski L., Lin Q., Matyjaszewski K., Schneider J.W., Tilton R.D. (2007). High capacity, charge-selective protein uptake by polyelectrolyte brushes. Langmuir.

[25] Lei H., Wang M., Tang Z., Luan Y., Liu W., Song B., Chen H. (2014). Control of lysozyme adsorption by pH on surfaces modified with polyampholyte brushes. Langmuir.

[26] Wang X., Berger R., Ramos J.I., Wang T., Koyonov K., Liu G., Butt H.J., Wu S. (2014). Nanopatterns of polymer brushes for understanding protein adsorption on the nanoscale. RSC Adv..

[27] vander Straeten A., Bratek-Skicki A., Jonas A.M., Fustin C.A., Dupont-Gillain C. (2018). Integrating Proteins in Layer-by-Layer Assemblies Indepenedently of Their Electrical Charge. ACS Nano.

[28] vander Straeten A., Bratek-Skicki A., Germain L., D‘Haese C., Eloy P., Fustin C.A., Dupont-Gillain C. (2017). Protein-Polyelectrolyte Compleaxes to Improve the Biological Acticity of Proteins in Layer-by-Layer Assemblies. Nanoscale.

[29] Bratek-Skicki A., Crisaudo V., Savocco J., Nootens S., Morsomme P., Delcorte A., Dupont-Gillian C. (2019). Mixed Polymer Brushes for the Selective Capture and Release of Proteins. Biomacromolecules.

[30] Bratek-Skicki A., Eloy P., Morga M., Dupont-Gillain C. (2018). Reversible protein adsorption on mixed PEO/PAA polymer brushes-role of ionic strength and PEO content. Langmuir.

[31] Välimäki S., Khakalo A., Ora A., Johansson L.S., Rojas O.J., Kostiainen M.A. (2016). Effect of PEG-PDMAEMA block copolymer architecture on polyelectrolyte complex formation with heparin. Biomacromolecules.

[32] Stamm M. (2008). Polymer Surfaces and Interfaces. Charact. Modif. Appl. Springer: Berl. Ger..

[33] Nicholson J.P., Wolmarans M.R., Park G.R. (2000). The role of albumin in critical illness. Br. J. Anaesth.

[34] Peters T., Stewart A.J. (2013). Albumin research in the 21st century. Biochim. Biophys. Acta.

[35] Adamczyk Z., Bratek-Skicki A., Żeliszewska P., Wasilewska M. (2014). Mechanisms of fibrinogen adsorption at solid substrates. Curr. Top. Med. Chem..

[36] Bratek-Skicki A., Zeliszewska P., Adamczyk Z. (2014). Human fibrinogen adsorption on latex particles at pH 7.4 studied by electrophoretic mobility and AFM measurements. Curr. Top Med. Chem..

[37] Bratek-Skicki A., Zeliszewska P., Adamczyk Ż. (2013). Tuning conformations of fibrinogen monolayers on latex particles by pH of adsorption. Colloids Surf. B Biointerfaces.

[38] Bratek-Skicki P., Zeliszewska J.M. (2016). Fibrinogen: A journey into biotechnology. Soft Matter.

[39] Blake C.C.F., Grace D.E.P., Johnson L.N., Perkins S.J., Phillips D.C., Cassels R., Dobson C.M., Poulsen F.M., Williams R.J.P., Porter R., Fitzsimons D.W. (1978). Physical and Chemical Properties of Lysozyme. Ciba Foundation Symposium 60—Molecular Interactions and Activity in Proteins.

[40] Li S., Mulloor J.J., Wang L., Ji Y., Mulloor C.J., Micic M., Orbulescu J., Leblanc R.M. (2014). Strong and selective adsorption of lysozyme on graphene oxide. ACS Appl Mater Interfaces.

[41] Regnier F.E., Cho W., Veenstra T.D. (2013). Affinity Targeting Schemes for Biomarker Research. Proteomic and Metabolomic Approaches to Biomarker Discovery.

[42] Goding J.W., Immunohistology (1996). Monoclonal Antibodies: Principle and Practice (Third Edition).

[43] Brandrup J., Immergut E.H., Grulke E.A., Abe A., Bloch D.R. (1989). Polymer Handbook.

