# Supplementary material for: Design of Ultra-Thin PEO/PDMAEMA Polymer Coatings for Tunable Protein Adsorption
Source: Polymers (Basel). 2020 Mar 15;12(3):660. doi: 10.3390/polym12030660 (PMC7183053; doi:10.3390/polym12030660)
Supplement: Supplementary file 1 [file polymers-12-00660-s001.pdf]

Supplementary Materials

Design of Ultra-Thin PEO/PDMAEMA Polymer Coatings for Tunable Protein Adsorption

Anna Bratek-Skicki [1,2]

[1] Institute of Condensed Matter and Nanosciences, Université catholique de Louvain, Place Louis Pasteur 1, bte L4.01.10, B-1348 Louvain-la-Neuve, Belgium; ncbratek@cyf-kr.edu.pl

[2] Jerzy Haber Institute of Catalysis and Surface Chemistry, Polish Academy of Sciences, Niezapominajek 8, PL30239 Krakow, Poland

1 Surface molar fractions of elements determined by XPS, decomposition of C1s peak and volumetric proportion of PEO and PDMAEMA

The following peaks (not shown) were detected by XPS: the N 1s signal was detected at 399 eV, the O 1s peak was recorded near 531.9 eV, while the S 2p peak was decomposed with two doublets, one near 163.2 eV assigned to unbound thiol/disulfide and another near 161.7 eV assigned to thiolate (i.e. attributed to the thiol function linked to gold)[1]. Next, two peaks representing the Au 4f doublet were found at 83 and 87 eV. The C 1s peak was recorded at ~ 285 eV and its decomposition is described below. Note that the gold substrate was detected at a significant level, so it is highly probable that the whole organic layer was probed. Detected gold fraction is lower for PDMAEMA in comparison to PEO1 and PEO2. It can be deduced that the coverage of the gold substrate is better for pure PDMAEMA brushes than for pure PEO brushes, except for PEO5. This exception is explained by the thicker layer formed by PEO5 in comparison to PEO1 and PEO2 owing to its longer polymer chains. In the same way atomic fractions (%) of gold and of the thiolate group (S-Au) for PEO1, PEO2, PEO5 decrease with increasing PEO molar mass.

Table S1. Surface composition determined by XPS (molar fractions in %).

Surface atomic fraction (%)	O	N	$\underline{C}$ = O	$\underline{C}$-(N,O)	$\underline{C}$-C=O	$\underline{C}$-(C,H)	C total	S total	Au total
Au-PDMAEMA	9.7	2.5	6.6	12.1	1.0	27.0	46.6	0.5	40.7
Au-PEO1 [2]	10.8	-	1.3	25.5	-	7.8	34.6	2.9	51.6
Au-PEO2 [2]	14.6	-	1.2	29.4	-	6.7	37.3	2.5	45.6
Au-PEO5[a] [2]	19.6	-	-	46.0	-	2.5	48.5	1.5	30.4
Au-PEO1/PDMAEMA $_{50/50}$	12.6	0.7	0.4	17.0	1.2	4.2	22.8	0.8	63.1
Au-PEO2/PDMAEMA $_{50/50}$	13.5	0.7	0.8	18.9	1.1	3.2	24.0	1.0	60.8
Au-PEO5/PDMAEMA $_{50/50}$	17.9	0.3	0.8	30.3	1.3	3.1	35.5	1.0	45.3
Au-PEO1/PDMAEMA $_{60/40}$	10.9	0.8	0.8	15.8	1.2	4.2	22.0	1.1	65.2
Au-PEO2/PDMAEMA $_{60/40}$	11.3	0.7	0.6	23.5	1.4	3.7	29.2	1.0	57.8
Au-PEO5/PDMAEMA $_{60/40}$	23.3	0.4	0.5	38.6	1.5	2.9	43.5	0.9	31.9
Au-PEO1/PDMAEMA $_{70/30}$	11.1	0.6	0.8	15.3	1.3	3.7	21.1	1.4	65.8
Au-PEO2/PDMAEMA $_{70/30}$	16.1	0.7	1.1	22.5	1.1	3.4	28.1	0.7	54.4
Au-PEO5/PDMAEMA $_{70/30}$	30.8	0.3	0.2	49.0	2.2	2.1	53.5	0.6	14.8

[a] - additionally 0.3 % of S (sulfates)

The C 1s peak of PEO (see Figure 3b-main text) was decomposed into three components: a) a characteristic $\underline{C}$-(N,O) component at 286.3 eV , b) $\underline{C}$-(C,H) at 284.8eV representing organic contaminants, c) a very small component at 288.8 eV representing more oxidized carbon present in the contamination. The C 1s peak of PDMAEMA (see Figure 2a-main text) was decomposed into the same components.
In order to obtain relative volumetric proportions of PDMAEMA and PEO in the layer probed by XPS, the following formula was used:

$$\left(\frac{\underline{C}-O-\underline{C}}{\underline{C}OOH}\right)_{mix}^{exp}=\frac{f_{vPEO}\times\left(\underline{C}-O-\underline{C}\right)_{PEO}^{exp}+f_{vPDMAEMA}\times\left(\underline{C}-O-\underline{C}\right)_{PDMAEMA}^{exp}}{f_{vPEO}\times\left(\underline{C}OOH\right)_{PEO}^{exp}+f_{vPDMAEMA}\times\left(\underline{C}OOH\right)_{PDMAEMA}^{exp}}$$

$$f_{vPEO}=1-f_{vPDMAEMA}$$

where $\left(\frac{\underline{C}-O-\underline{C}}{\underline{C}OOH}\right)_{mix}^{exp}$ is the experimental area ratio of the C 1s components measured at 286.3 eV and 288.8 eV in the mixed brushes. $\left(\underline{C}-O-\underline{C}\right)_{PEO}^{exp},\left(\underline{C}-O-\underline{C}\right)_{PDMAEMA}^{exp}$ are the areas of the C 1s component measured at 286.3 eV assigned to the PEO and PDMAEMA homobrushes. $\left(\underline{C}OOH\right)_{PEO}^{exp}$ and $\left(\underline{C}OOH\right)_{PDMAEMA}^{exp}$ are the areas of the C 1s component measured at 288.8 eV corresponding to the PEO and PDMAEMA homobrushes. The $\underline{C}$-O-$\underline{C}$ fraction of PEO in the mixed brushes was calculated as the result of the following substraction of atomic concentration (%)
$(\underline{C}\text{-}O\text{-}\underline{C})_{PEO} = (\underline{C}\text{-}O\text{-}\underline{C})_{Total} - \underline{C}{=}O) - 3x(N)$

The relative mass proportions of PEO and PDMAEMA in the mixed brushes were then calculated using the density of each polymer, i.e., 1.13g/cm^3 and 1.32 g/cm^3 [3] for PEO and PDMAEMA, respectively. It should be noted that the equations were used assuming a homogenous distribution of both polymers in the analyzed volume.

2. AFM images of created coatings

The roughness of the gold surface is clearly observed, visualizing gold grains. Therefore, it can be concluded that the polymer layer is very thin, and no aggregation can be observed.

Table S2. AFM images of PEO, PDMAEMA and PEO/PDMAEMA70/30 brushes created on a gold substrate.

AFM images	
Au 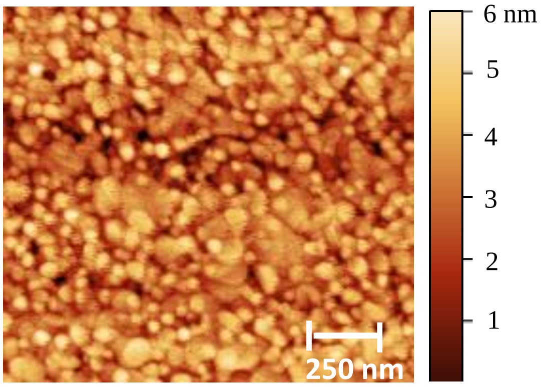	Au-PDMAEMA 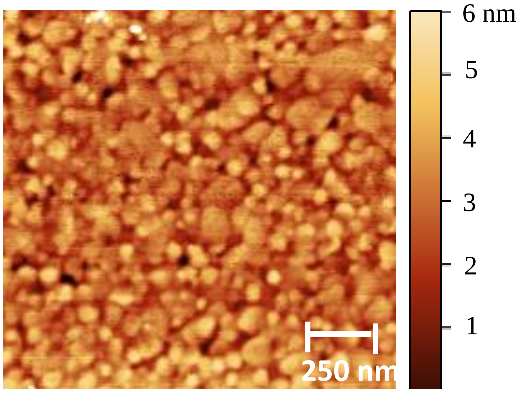

Table S2. *Cont.*

Au-PEO1<br>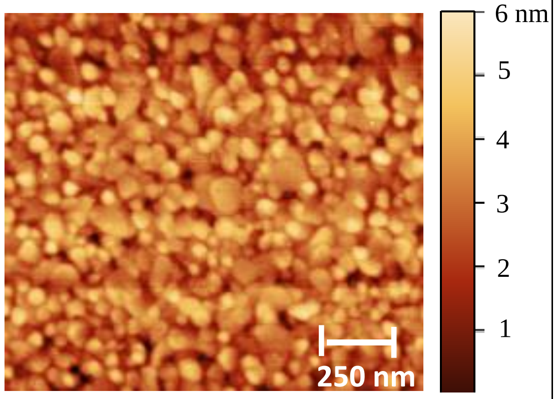	Au-PEO1/PDMAEMA 70/30<br>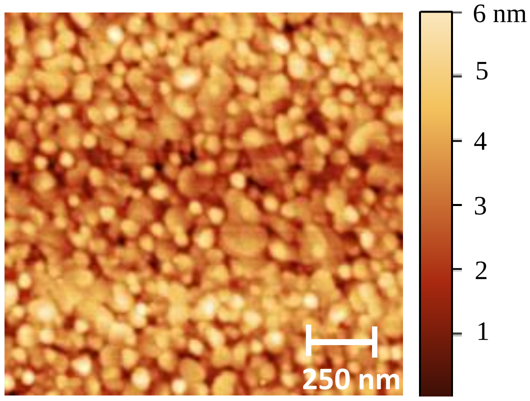
Au-PEO2/PDMAEMA 70/30<br>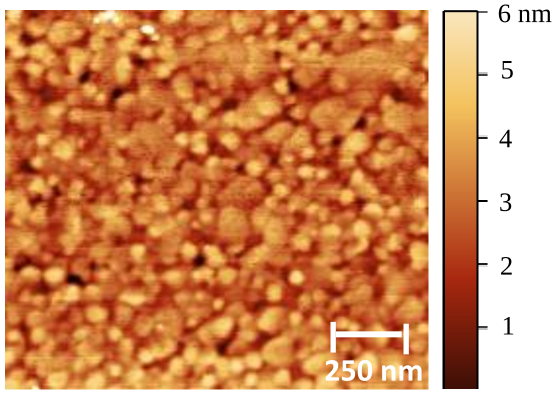	Au-PEO5/PDMAEMA 70/30<br>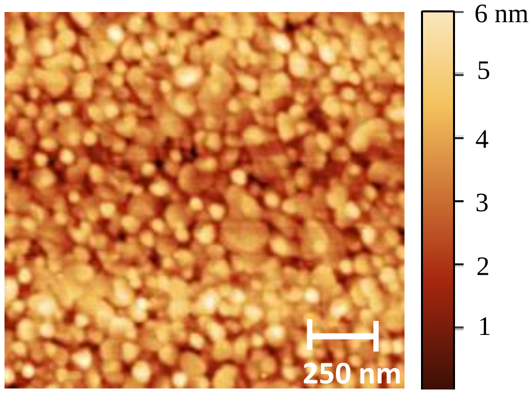

3. Protein-repellent properties of PEO coating

A typical QCM experiment illustrating the protein repellent properties of PEO is presented in Figure 1.S. After stabilization under water, the PEO5 solution was flowed into the cell, allowing a frequency shift corresponding to the polymer coating formation to be measured. The frequency shift stabilizes and does not change much during rinsing with pure water. The next shift corresponds to the conformational changes of PEO after the introduction the saline solution (I=10^{-3}M, pH 7.4). After flowing the Fb solution, no frequency shift was observed, indicating the lack of adsorption, in accord with the expected protein-repellent properties of PEO.

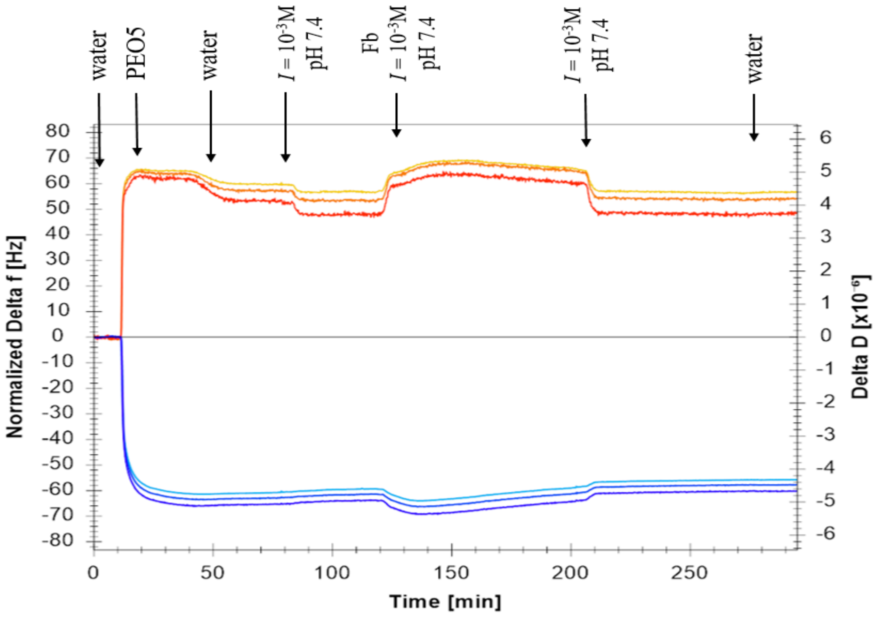

Figure S1. Example of a representative QCM experiment: PEO5 polymer coating formation and adsorption of Fb at I =10^{-3}M, pH 7.4.

4. Adsorption of Lys and Av on the PEO1/PDMAEMA 70/30 coatings and cyclic adsorption/desorption of HSA on the PEO5/PDMAEMA 70/30 coating.

A series of experiments showing the adsorption of Lys and Av were performed on the pure and the mixed PEO/PDMAEMA coatings. Two examples representing the adsorption of Lys and Av on the PEO1/PDMAEMA 70/30 are shown in Figures 2.S and 3.S, respectively.

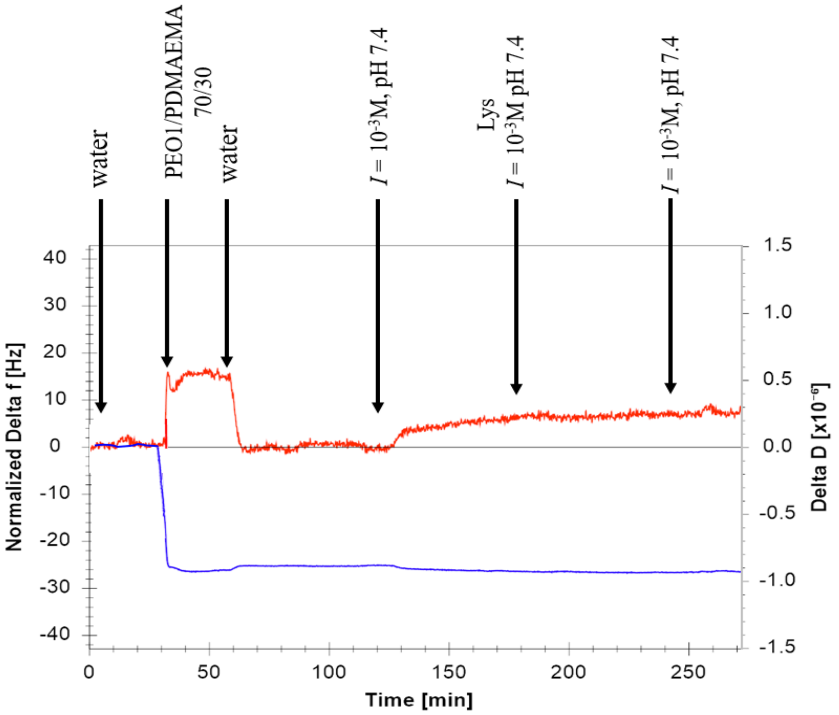

Figure S2. Example of a representative QCM experiment: PEO1/PDMAEMA 70/30 polymer coating formation and adsorption of Lys at I =10^{-3}M, pH 7.4.

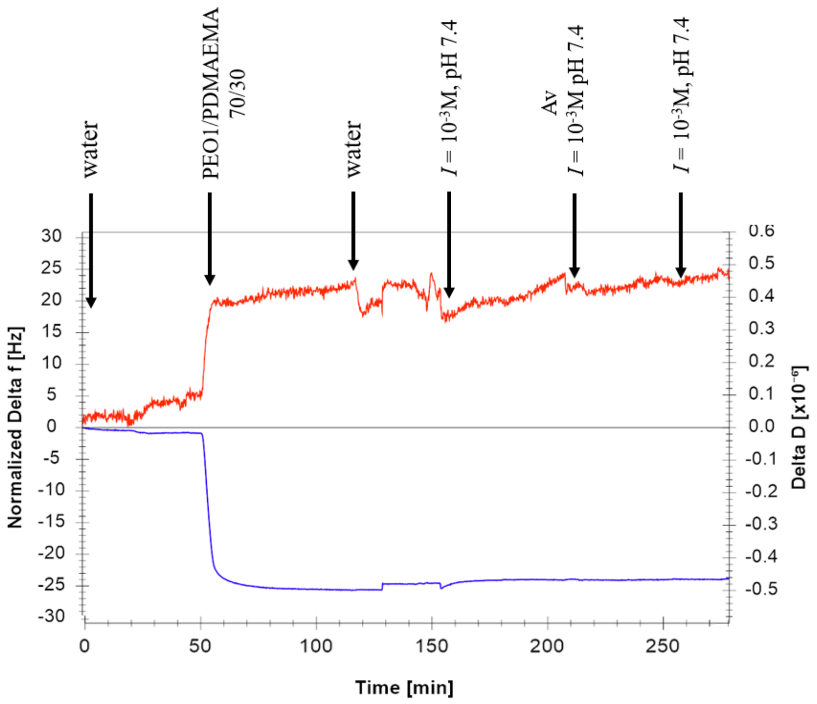

Figure S3. Example of a representative QCM experiment: PEO1/PDMAEMA 70/30 polymer coating formation and adsorption of Av at I =10^{-3}M, pH 7.4, showing the fifth overtone.

After the polymer coating formation, Lys or Av solution was introduced into the cell and no changes in frequency was observed indicating a lack adsorption of both polymers. Similar results were observed for the pure and the mixed coatings

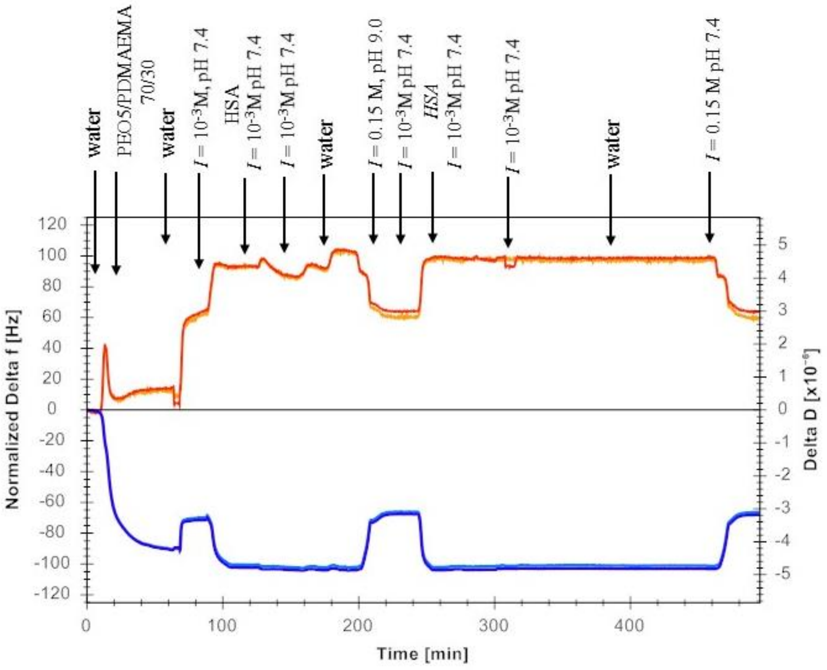

Figure S4. Example of a representative QCM experiment showing the grafting of the polymers (PEO5/PDMAEMA 70/30), followed by 2 cycles of adsorption/desorption of HSA at I =10^{-3}M, pH 7.4.

References

[1] D.G Castner, K. Hinds, D.W. Grainger, X-ray photoelectron spectroscopy sulfur 2p study of organic thiol and disulfate binding interactions with gold surfaces, *Langmuir*, **1996**, *12*, 5083-5086.

[2] A. Bratek-Skicki, P. Eloy, M. Morga, C. Dupont-Gillain, Reversible Protein Adsorption on Mixed PEO/PAA Polymer Brushes: Role of Ionic Strength and PEO Content, *Langmuir* 2018, *34*, 3037-3048.

[3] L. Brandrup, E. H. I., E.A. Grulke, *Polymer Handbook*. Wiley & Sons: Hoboken, NJ, USA, 1999.
